# A seasonal aggregation of juvenile scalloped hammerheads *Sphyrna lewini* along beaches of the Gold Coast, Australia

**DOI:** 10.1111/jfb.70118

**Published:** 2025-06-18

**Authors:** Nicolas Lubitz, Kerryn Doupain, Siobhan Houlihan, Jonathan D. Mitchell

**Affiliations:** ^1^ Biopixel Oceans Foundation Cairns Queensland Australia; ^2^ College of Science and Engineering James Cook University Townsville Queensland Australia; ^3^ Department of Primary Industries Queensland Boating and Fisheries Main Beach Queensland Australia; ^4^ Sea World Main Beach Queensland Australia; ^5^ Queensland Government Department of Primary Industries Dutton Park Queensland Australia

**Keywords:** coastal habitats, elasmobranchs, hammerheads, nursery

## Abstract

We document a recurring seasonal aggregation of juvenile scalloped hammerhead sharks *Sphyrna lewini* along the beaches of the Gold Coast, Australia, with notable occurrences at Burleigh Heads and Kirra Reef. The aggregation consists of individuals of varying sizes, which likely suggests the presence of different cohorts, though it remains uncertain whether this area meets the criteria for a nursery habitat. Given the global population declines of *S. lewini*, its classification as Critically Endangered by the IUCN and its Conservation Dependent status under Australian legislation, we recommend standardised surveys and tracking studies to assess habitat use, seasonality and the potential role of Gold Coast waters as a critical habitat for juvenile *S. lewini*.

Many mobile marine species rely on a variety of critical habitat types to support key functions throughout life history (Amorim et al., 2018; Barnett et al., 2019; Dahlgren & Eggleston, 2000; Persson & Crowder, 1998; Sheaves et al., 2015). In elasmobranchs (sharks and rays), the dependence on disparate habitats of juveniles and adults is frequently reported (Chin et al., 2013; Grubbs, 2010; Heupel et al., 2018; Knip et al., 2011; Martins et al., 2018). For instance, the utilisation of specific nurseries, separate from adults, appears to be an important survival strategy for juveniles of many species (Castro, 1993; Heithaus, 2007; Heupel et al., 2007). Often, such nurseries consist of shallow, inshore habitats that are believed to offer ample resources needed for growth and/or protection from predators, including conspecifics (Duncan & Holland, 2006; Heupel & Simpfendorfer, 2011; Stump et al., 2017).

The scalloped hammerhead *Sphyrna lewini* (Griffith & Smith 1834) is a highly mobile, viviparous species that occurs in tropical and subtropical areas worldwide (Ebert & Fowler, 2021). Because of overfishing and degradation of coastal habitats, the species is currently listed as Critically Endangered globally (Rigby et al., 2019) and as Conservation Dependent under Australia's EPBC Act (EPBC, Australian Government, 1999). Adult and juvenile *S. lewini* are known to form predictable, recurring seasonal aggregations at key locations (Bessudo et al., 2011; Hearn et al., 2010; Klimley et al., 1988; López et al., 2023). Although records of mature females are rare, they are believed to primarily occupy offshore, pelagic habitats, while males tend to remain inshore (Harry et al., 2011; Hoyos‐Padilla et al., 2014; Wells et al., 2018). During the warmer summer months, pregnant females likely migrate inshore and give birth to litters ranging from nine to 23 pups in Australian waters with a size at birth between 45 and 56 cm total length (TL) (Harry et al., 2011; Stevens & Lyle, 1989). Neonates and juveniles occur in shallow, turbid inshore nurseries and likely reside there for at least several months (Brown et al., 2016; Simpfendorfer & Milward, 1993; Yates et al., 2015; Zanella et al., 2019). However, seasonal habitat selection of juveniles, residency times in nurseries and movements after leaving nursery areas remain poorly understood (Duncan & Holland, 2006; Hutchinson et al., 2023; Simpfendorfer & Milward, 1993). The objectives of this study were to (1) provide an initial description of a recently discovered aggregation of juvenile *S. lewini* along surf beaches of the Gold Coast, Queensland, Australia and (2) discuss recommendations for future research to determine the area's role as an essential habitat.

The Gold Coast is situated along the subtropical coast of southeast Queensland, Australia (Figure 1a). It is a popular tourism hotspot and famous for its sandy beaches and favourable surfing conditions. In recent decades, the area has become highly populated and urbanised (Hundloe et al., 2015; Treby & Castley, 2016). Because of the influence of well‐mixed oceanic waters, this inshore region is defined by reduced turbidity, which only increases during windy conditions or after heavy rainfall as freshwater discharges through estuaries, namely the Tweed River, Currumbin Creek and Tallebudgera Creek (Figure 1b). The waters of the area have an average temperature of 19°C during the austral winter and 24°C during the austral summer (Pattiaratchi & Pattiaratchi & Hetzel, 2020). Here, among other parts of the Queensland coast, the Queensland state government installed a bather protection programme in the 1960s to remove potentially dangerous shark species, mainly white sharks *Carcharodon carcharias* (Linnaeus, 1758), tiger sharks *Galeocerdo cuvier* (Péron & Lesueur 1823) and bull sharks *Carcharhinus leucas* (Müller & Henle 1839), from inshore waters, consisting of baited drum lines and shark nets, called the Queensland Shark Control Program (QSCP) (Figure 1a). Catch data from this program is publicly available and can be accessed for scientific studies, for example.

**FIGURE 1:**
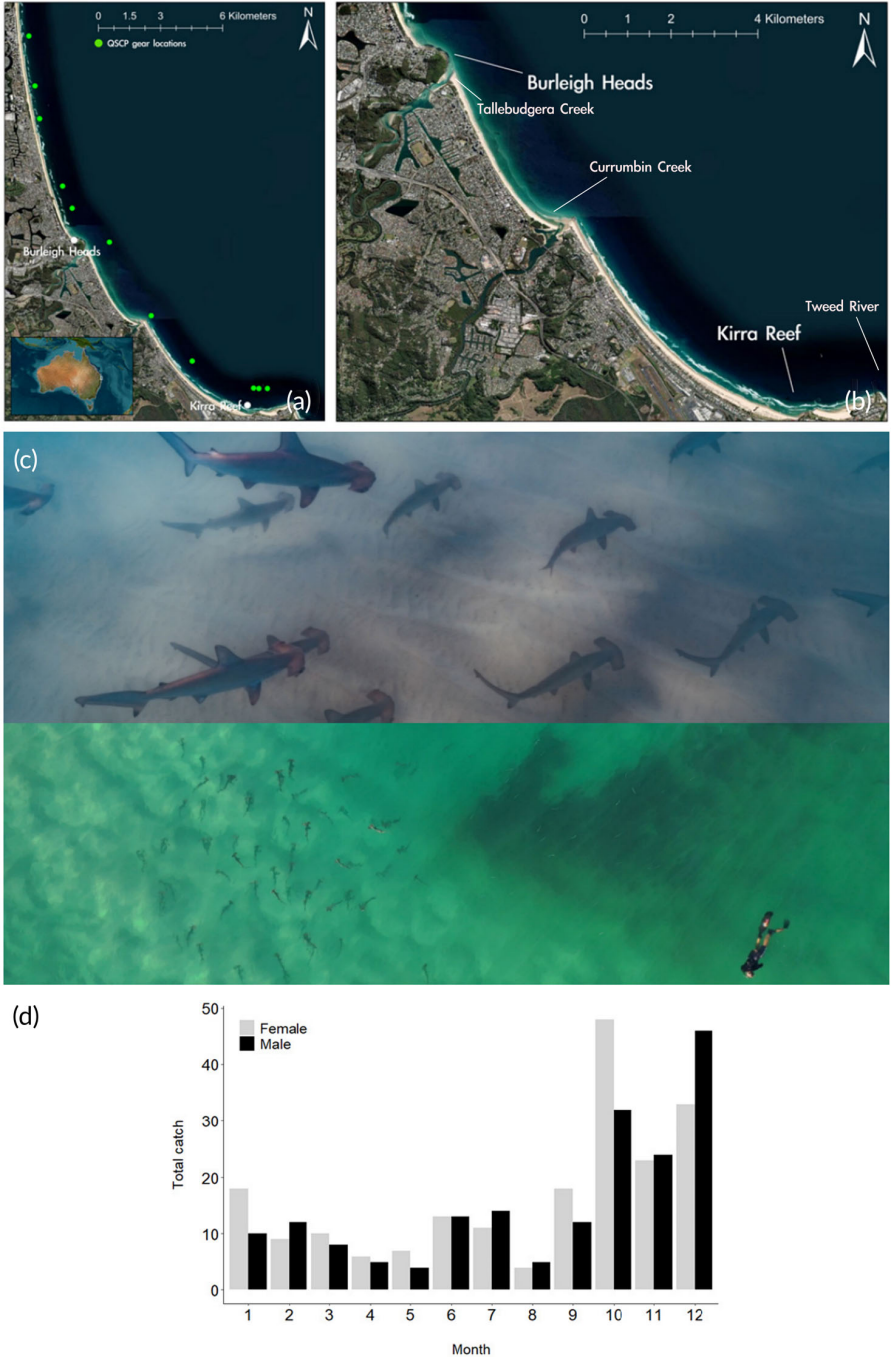
(a) Map of the Gold Coast, Queensland, Australia. (a) The locations of Queensland Shark Control Program gear on which mature female (>190 cm) and juvenile *S. lewini* (<120 cm) were caught. (b) Enlarged view of Burleigh Heads and Kirra Reef, where aggregations of juvenile *S. lewini* have been observed. (c) Top: An underwater photo of juvenile *S. lewini* aggregating at Burleigh Heads on the 4th of April 2024. Photograph credit: Tiani Dun. Bottom: Screenshot from an aerial drone video of the aggregation at Burleigh Heads, showing the different size classes in relation to snorkellers. Photograph credit: Blaze Parsons. (d) Catch data for nets and drumlines of the QSCP deployed along Gold Coast beaches from 2000 to 2024, total number of *S. lewini* caught per month (juveniles and adults).

In March 2023, recreational freedivers and surfers first reported to the authors a large aggregation of juvenile *S. lewini* in shallow, clear waters off the beach at Burleigh Heads (Figure 1b). To confirm species identification, we used the same characteristics outlined in López et al. (2023). The aggregation quickly gathered significant media attention and became a local attraction for snorkellers and freedivers. Although informal and not representing standardised methodology, we aimed to review available information to gain initial insights into the spatio‐temporal dynamics of this aggregation. This included social media records, news outlets, drone videos supplied by members of the public, anecdotal communications with members of the public who reached out to the authors and underwater video recordings from non‐standardised free dives undertaken by the authors. For example, the authors regularly accessed news outlets and social media reports while remaining in contact with local snorkellers to determine when the aggregation was first reported in a given year and when reports ceased that same year. Permission to use videos and personal observations for analysis was sought from all members of the public.

Following social media and news reports, the shallow waters off Burleigh Heads were monitored through irregular freedives conducted by the authors and members of the public who forwarded their sightings to the authors during March and April 2023. Underwater visibility at the site was estimated to vary between 3 and 10 m throughout the period. Juvenile *S. lewini* aggregations were observed and documented on 12 occasions, with each observation lasting a minimum of 30 min. However, by mid‐April 2023 deteriorating sea conditions due to increased wind, rainfall and swell significantly reduced underwater visibility and discouraged further in‐water observations. No further additional sightings were reported through news outlets/social media and freediving surveys at Burleigh Heads once conditions improved later in the year.

Beginning in late April 2023, fishers operating at Kirra Reef, approximately 10 km south of Burleigh Heads, reported to the authors continued sightings and incidental captures of juvenile *S. lewini* through to at least June and July. Based on personal communication with a fisher with over 20 years of experience working at Kirra Reef, juvenile *S. lewini* have occurred at the site for many years, including individuals less than 100 cm in total length during May and June 2023. However, unfavourable sea conditions, particularly strong surf, prevented freediving at Kirra Reef during this time.

In 2024, the first sightings of juvenile *S. lewini* at Burleigh Heads were retrieved from news outlets and were directly communicated to the authors by members of the public in March, with observations ceasing again by April. Irregular freedives documented the aggregation on 10 occasions under favourable conditions, with each underwater observation lasting a minimum of 30 min. A few days of favourable visibility allowed the review of both drone and underwater footage supplied by members of the public, indicating an estimated ~50 individuals at the site. Subsequent reports of an aggregation at Kirra Reef, approximately 10 km south of Burleigh Heads, indicated the presence of juvenile *S. lewini* until at least late June 2024 (Table 1). Unlike the previous year, diving conditions permitted direct underwater observations at Kirra Reef, with freedivers recording *S. lewini* on 14 occasions in May and June, as reported to the authors. Fishers also reported juvenile sightings during this period to the authors. Although, the methods used here were not applied past 2024, freedivers reported sightings to the authors occurring in January/February 2025 at Kirra Reef, extending the annual timeframe for observations of juvenile *S. lewini*. This indicates juveniles may aggregate in this area for at least 6 months, but standardised surveys are needed to resolve the temporal dynamics.

**TABLE 1:** Summary of 2 years of drone and freediving observations on the aggregation of juvenile *S. lewini* along Gold Coast beaches.

Year	Duration of sightings	Locations of sightings	Size classes observed	Habitat	Size of aggregation
2023	March to April	Burleigh Heads beach	~50–120 cm	Depth: 3–6 m Substrate: sand Other dominant species: small teleost fish and common stingarees *Trygonoptera Testacea* (Müller & Henle 1841) Nearby river mouths: Currumbin Creek and Tallebudgera Creek	Up to 50 individuals
2024	March to June/July	Burleigh Heads beach and Kirra Reef	~50–120 cm	For Burleigh Heads beach see row above Kirra Reef: Depth: 6–12 m Substrate: sand adjacent to rocky reef (including brown algae, soft and hard coral) Other dominant species: small teleost fish Nearby river mouths: Tweed River	>50 individuals

The seasonal timing of aggregations in both 2023 and 2024 closely aligns with that of a known juvenile *S. lewini* aggregation in the Rewa River estuary, Fiji, which occurs between February and June in a tropical context (Brown et al., 2016). Notably, estuarine systems are also adjacent to both Burleigh Heads and Kirra Reef, where the immediate habitat consists of shallow sandy bottoms and gutters near rocky outcrops (Figure 1). It remains unclear if all, some or none of the individuals sighted at Burleigh Heads in April of both 2023 and 2024 take part in the aggregation at Kirra Reef. Observations from fishers and water users suggest that this aggregation may migrate south from Burleigh Heads to Kirra and may be a persistent annual, but seasonal phenomenon. However, more standardised surveys (Mitchell et al., 2022) and possible tagging studies are needed to confirm the connection between sightings at Burleigh Heads and Kirra Reef.

Although the length of individuals could not be determined with any accuracy, based on our observations and using the size of snorkelers/surfers for approximations, the aggregation consists of neonates and larger juveniles (~50–120 cm TL) and includes multiple cohorts (Figure 1c). Photographic evidence from underwater encounters and drone imagery suggests at least 50 individuals can be present at times at both Burleigh Heads and Kirra Reef. However, underwater encounters by the public as well as the authors of this study demonstrate that the number of aggregating individuals can change from day to day, with group sizes possibly exceeding 50 individuals but also ranging down to less than five individuals (Table 1).

Overall, the frequency and reliability of sightings in both 2023 and 2024 appeared directly related to water clarity and weather conditions. This may explain why no individuals were reported from Kirra Reef in 2023. Kirra Reef consists of scattered rocky outcrops adjacent to sandy bottom habitat and is situated approximately 400 m offshore from the Kirra surf break. Because of local oceanography, access by snorkellers and spear fishers, and thus the possibility of observing *S. lewini*, is heavily dependent on weather and sea conditions. This contrasts with the shallower, more protected waters inside Burleigh Heads. Thus, persistent, challenging conditions could have prevented sightings from Kirra in 2023. Fishers who access Kirra Reef via boat have regularly reported catching and releasing juvenile hammerheads over the years. Despite initial inferences, standardised survey efforts are needed to determine the exact spatio‐temporal dynamics of this aggregation and resolve habitat selection choices.

The total catch data of *S. lewini* recorded in shark nets and on baited drum lines deployed by the QSCP along Gold Coast beaches from 2000 to 2024 indicate a seasonal increase in the abundance of both male and female individuals (combined size classes) between October and December (Figure 1d). Catches of juvenile *S. lewini* (<120 cm total length) were generally low, occurring primarily between September and February (Queensland Department of Agriculture and Fisheries). The shark nets used in the QSCP feature large mesh sizes (50 × 50 cm) and are not designed to target small‐bodied sharks; however, catch data indicate an increase in larger females (>190 cm total length) between November and January, coinciding with the peak pupping season (Harry et al., 2011; Stevens & Lyle, 1989). These findings further support the hypothesis that mature females primarily inhabit offshore waters outside the reproductive season and migrate inshore for mating and parturition (Harry et al., 2011; Hoyos‐Padilla et al., 2014; Klimley, 1987; Stevens & Lyle, 1989). Notably, on 31 December 2020, a pregnant female was captured in a shark net off Coolangatta beach, containing nine near‐term pups (<50 cm total length), further suggesting inshore parturition.

Potential habitat shifts are apparent in nurseries in Costa Rica, Florida and Hawaii (USA) (Hutchinson et al., 2023; Wargat et al., 2024; Zanella et al., 2019). Here, although exhibiting high residency over small spatial scales for up to 5 months, juveniles disappear from the respective study areas for the rest of the year, with no data on their whereabouts, then return the following year (Hutchinson et al., 2023; Wargat et al., 2024; Zanella et al., 2019). Additionally, the highest catches of juvenile *S. lewini* in a tropical, turbid bay in northern Queensland occurred from February to July, outside the possible peak pupping season, suggesting that young‐of‐the‐year in North Queensland move away from immediate natal habitats within months after birth and seasonally select specific habitats (Simpfendorfer & Milward, 1993).

Overall, habitat selection choices in juvenile *S. lewini* retain knowledge gaps, specifically which habitat types are selected at different times of the year and why (Duncan & Holland, 2006; Hutchinson et al., 2023; Simpfendorfer & Milward, 1993; Yates et al., 2015). Long‐term catch data in northern Florida (USA) indicate that juveniles are locally abundant during the pupping season in the Tolomato River mouth but not adjacent river systems, suggesting selective seasonal habitat choices as reported elsewhere (Bessudo et al., 2011; Hearn et al., 2010; Klimley et al., 1988; López et al., 2023; Wargat et al., 2024). Additionally, catches in multiple bays in northern Queensland indicate that juveniles do not occur evenly across sampled bays; instead, catches were highest with warmer temperatures and in highly turbid bays (Yates et al., 2015). Habitat use of juveniles is often linked to warm, turbid waters close to estuaries (Yates et al., 2015). In contrast, although habitats in which juveniles aggregate along the subtropical Gold Coast are near estuaries, they consist of less turbid, sandy areas adjacent to rocky reefs with predominating marine influence (except for during major flood events), where temperatures during the austral winter can fall to as low as 16°C (Integrated Marine Observing System, 2024). Thus, the role of environmental variables driving seasonal habitat selection in juvenile *S. lewini* remains unclear and may be context‐dependent (López et al. 2023; Lubitz et al., 2022; Wargat et al., 2024).

Long‐term studies from a confirmed nursery in Kane'ohe Bay, O'ahu, Hawaii demonstrated that juveniles, especially neonates, are often in poor nutritional condition, causing relatively high mortality through starvation (Duncan & Holland, 2006). This indicates that the necessity to avoid predators likely outweighs the limited availability of prey resources in this nursery (Duncan & Holland, 2006). Determining whether resource availability, predator avoidance or other ecological factors drive the seasonal aggregation of juvenile *S. lewini* in potential nursery areas along the Gold Coast requires further investigation. However, quantifying these drivers remains challenging and is poorly understood for many species and across diverse geographical regions (Heupel et al., 2007; Heupel et al., 2018; Martins et al., 2018). The recent establishment of an extensive acoustic tracking array along the Queensland coast, including receivers on the Gold Coast and long‐term tracking projects that have already acoustically tagged large, mobile shark species along the coast, would facilitate the study of overlap between juvenile *S. lewini* and potential predators at this aggregation site (Barnett et al., 2022; Barnett et al., 2024). Thus, we recommend the tagging of juveniles and deployment of additional acoustic receivers along Gold Coast beaches and inshore waterways to the determine the role of predator avoidance in selecting shallow, inshore habitats.

For an area to classify as a nursery, it must meet three widely accepted criteria: (1) juveniles occur more frequently in the area than in other locations where the species is generally found; (2) juveniles exhibit site fidelity and remain in the area for extended periods; and (3) the area is used consistently across multiple years (Heupel et al., 2007). While our findings suggest that juvenile *S. lewini* aggregate in shallow inshore waters between Burleigh Heads and Kirra Reef for possibly 6 months and exhibit site fidelity, the exploratory and descriptive nature of our study precludes definitive conclusions. Furthermore, we were unable to assess whether juveniles occur more frequently in our study area compared to other habitats, preventing a full evaluation of nursery criteria. Future research, including catch surveys, drone‐based monitoring and tracking studies, is essential to determine whether this region functions as a nursery and to enhance our understanding of spatiotemporal habitat use in juvenile *S. lewini* along Australia's east coast. Given the species' conservation status and the proximity of this aggregation to QSCP gear, as well as to heavily urbanised and recreationally used areas, addressing these knowledge gaps is a priority for effective conservation and management.

## AUTHOR CONTRIBUTIONS

N.L., J.D.M and K.D. conceived the study. N.L. wrote the manuscript. S.H. provided critical insights on aspects of the hammerhead aggregation. All authors reviewed, edited and approved the final manuscript.
